# Urban slum structure: integrating socioeconomic and land cover data to model slum evolution in Salvador, Brazil

**DOI:** 10.1186/1476-072X-12-45

**Published:** 2013-10-20

**Authors:** Kathryn P Hacker, Karen C Seto, Federico Costa, Jason Corburn, Mitermayer G Reis, Albert I Ko, Maria A Diuk-Wasser

**Affiliations:** 1 Department of Epidemiology of Microbial Disease, Yale School of Public Health, 60 College St, New Haven, CT 06511, USA; 2 School of Forestry and Environmental Studies, Yale University, 195 Prospect St, New Haven, CT 06511, USA; 3 Centro de Pesquisas Gonçalo Moniz, Fundação Oswaldo Cruz, Ministério da Saúde, Salvador 40296-710, Brazil; 4 Department of City and Regional Planning & School of Public Health, University of California Berkeley, Wurster Hall, Berkeley, CA 94720, USA

## Abstract

**Background:**

The expansion of urban slums is a key challenge for public and social policy in the 21^st^ century. The heterogeneous and dynamic nature of slum communities limits the use of rigid slum definitions. A systematic and flexible approach to characterize, delineate and model urban slum structure at an operational resolution is essential to plan, deploy, and monitor interventions at the local and national level.

**Methods:**

We modeled the multi-dimensional structure of urban slums in the city of Salvador, a city of 3 million inhabitants in Brazil, by integrating census-derived socioeconomic variables and remotely-sensed land cover variables. We assessed the correlation between the two sets of variables using canonical correlation analysis, identified land cover proxies for the socioeconomic variables, and produced an integrated map of deprivation in Salvador at 30 m × 30 m resolution.

**Results:**

The canonical analysis identified three significant ordination axes that described the structure of Salvador census tracts according to land cover and socioeconomic features. The first canonical axis captured a gradient from crowded, low-income communities with corrugated roof housing to higher-income communities. The second canonical axis discriminated among socioeconomic variables characterizing the most marginalized census tracts, those without access to sanitation or piped water. The third canonical axis accounted for the least amount of variation, but discriminated between high-income areas with white-painted or tiled roofs from lower-income areas.

**Conclusions:**

Our approach captures the socioeconomic and land cover heterogeneity within and between slum settlements and identifies the most marginalized communities in a large, complex urban setting. These findings indicate that changes in the canonical scores for slum areas can be used to track their evolution and to monitor the impact of development programs such as slum upgrading.

## Background

The UN-Habitat estimates that between 800 million to a billion people live in urban slums [1]. The rapid growth of urban slums, particularly in resource poor countries, is a critical challenge for social and public policy in the 21^st^ century [1]. In 2003, the UN- HABITAT operationally defined urban slum communities as areas that possess inadequate access to safe water, inadequate access to sanitation and other urban infrastructure, poor structural quality of housing, overcrowding, and insecure residential status, or a subset of these characteristics [1,2]. This operational definition was formulated in part to enable better accounting and monitoring of urban slums at a global scale, but offers limited guidance for local policy and interventions.

The inherent heterogeneity and dynamic nature of urban slums is a barrier to efforts aimed at creating a unified slum definition. Existing operational definitions, such as the UN- HABITAT criteria, do not capture the degree of infrastructure deficiencies and deprivation between and within slum communities: an area lacking secure access to tenure is classified the same as an area lacking piped water, sanitation, and durable housing materials [3,4]. Efforts to map slum location and extent are further limited by the lack of a direct association between land cover features typically associated with slums and levels of socioeconomic deprivation. Rather, gradations in standards of living exist even within slums, amplifying health disparities and creating hotspots of the transmission of infectious disease, as well as non-communicable health problems [4,5]. A more nuanced characterization of slums as continuous - rather than dichotomous, entities is necessary to capture the socioeconomic gradients within complex urban settings and the heterogeneity within and among slum communities.

New approaches that capture the multidimensional structure of urban slums are required to guide policy decisions, evaluate the outcome of slum upgrading interventions, and monitor the evolution of slum communities. Although it is well established that slums are significantly heterogeneous across both land cover and socioeconomic dimensions, and the UN-Habitat, Cities Alliance, and some NGOs have the goal to integrate this heterogeneity [6-8], implementation of integrated policies remains a challenge. Previous studies that have focused on mapping urban slums have relied either on geocoding land cover metrics such as the texture and shape of structures [9-13] or socioeconomic variables aggregated to the census level [3,14-16]. For a truly integrative approach, we need to address the interrelatedness between the land cover and socioeconomic deprivation factors which reflect the current and historical social and health policies that shaped the structure of a specific slum area.

In this study, we tested the hypothesis that land cover characteristics are associated with socioeconomic characteristics in Salvador, a city of 3 million inhabitants in Northeast Brazil [17]. To assess this hypothesis we modeled the interrelationship between socioeconomic and land cover characteristics of urban communities using canonical correlation analysis, a multivariate statistical technique commonly used in ecology [18]. As a methodological exercise, we used metrics derived from the canonical correlation analysis to map the multidimensional structure of slum settlements in Salvador. In doing so, we derived a continuous metric representing gradients in standards of living and deprivation, which we used to map the communities at 30 m × 30 m resolution. The resulting map captured the heterogeneity and spatial extent of slums and provided insights into the evolution of urban slums in Salvador, Brazil. While our approach focused on one city, the methods may be relevant and applicable for the challenges of analyzing spatial variation in urban informal settlements across Latin America, Asia and Africa [19].

## Results

### Approach overview

**Figure 1:**
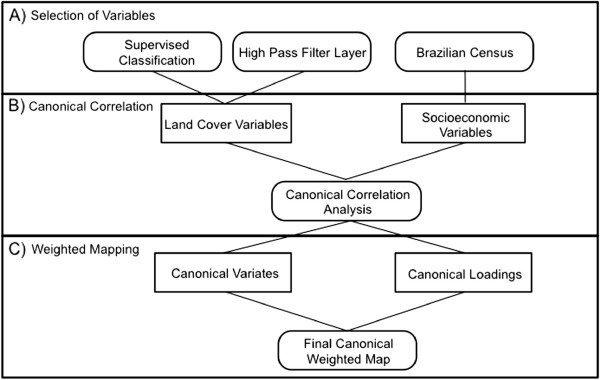
**Flow diagram of general approach.** Flow diagram describing the general approach for creating a final map of deprivation in Salvador. The approach is composed of three main steps: **A)** Selection of variables, **B)** Canonical correlation analysis and **C)** Mapping using weights from the canonical correlation analysis.

We used canonical correlation analysis to model the association between sets of variables characterizing the land cover and socioeconomic urban features in Salvador, Brazil, and applied this association to map slums at a higher spatial resolution than the smallest census unit (Figure 1). Land cover and socioeconomic variables were derived from a 2002 land cover classification and the 2000 Brazilian census, respectively (Figure 1A). Canonical correlation analysis identified a set of orthogonal axes that maximized the correlation between the land cover and socioeconomic variable sets (Figure 1B). The first two canonical dimensions were used to map deprivation across Salvador, Brazil, at a 30 m × 30 m resolution, using the canonical loadings as weights to reflect the importance of each variable in the overall urban structure (Figure 1C).

### Socioeconomic variable selection

We evaluated a number of variables in the 2000 Brazilian census associated with the UN-HABITAT slum definition, in particular those related to income (e.g. proportion of households with female head, average income of heads of household), education (e.g. years of education completed, proportion of heads of household that are illiterate), and infrastructure (e.g. proportion of households without a bathroom, proportion of households with piped water) (Table 1). From this list, we selected a subset of variables that had a linear relationship with all of the land cover variables (Table 2), which is a requirement of the canonical correlation analysis. We assessed the linearity of this relationship by visually assessing linear trends in a scatter plot matrix of all socioeconomic variables against all percent land cover types.

**Table 1:** Descriptive statistics of all socioeconomic characteristics considered across all census tracts

**Census variables**	**Minimum**	**1st quartile**	**Median**	**Mean**	**3rd quartile**	**Maximum**
**Proportion of households:**						
Without a bathroom*	0.000	0.000	0.006	0.022	0.018	0.598
Without garbage collection	0.000	0.964	0.998	0.938	1.000	1.000
Where garbage is thrown on an empty lot or street*	0.000	0.000	0.000	0.044	0.017	0.902
With more than 6 residents*	0.000	0.112	0.154	0.147	0.186	0.400
Connected to a sewer	0.000	0.004	0.023	0.154	0.168	1.000
With piped water *	0.000	0.910	0.972	0.923	0.996	1.000
With female head of household	0.011	0.320	0.376	0.375	0.430	0.747
**Proportion of heads of households:**						
With more than 8 years of education	0.040	0.344	0.500	0.526	0.725	0.968
That are illiterate	0.000	0.015	0.062	0.075	0.115	0.511
**Census tracts where the:**						
Average income of heads of households per month*	99.570	286.200	421.500	833.9	893.800	7978.000
Population density	0.000	210500	589800	1126000	1239000	124900000

*Variables that met the linearity requirement and met the UN-HABITAT definition for urban slum.

**Table 2:** Socioeconomic and land cover variables used in the canonical correlation analysis

**Variables**	**Description slum characteristics**
**Socioeconomic**	
Bathroom	Proportion of households without bathroom
Water	Proportion of households with piped water
Garbage	Proportion of households where garbage thrown on an empty lot or street
Income	Average income of heads of household per month
Crowding	Proportion of households with more than 6 residents
**Land cover**	
Corrugated roof	Proportion of corrugated roofs per impervious surfaces within a census tract
White-painted roof	Proportion of white-painted roofs per impervious surfaces within a census tract
Tile roof	Proportion of tile roofs per impervious surfaces within a census tract
Pavement	Proportion of pavement per impervious surfaces within a census tract
Texture	Standard deviation of the high-pass filter of the red band within a census tract

### Land cover variable derivation

**Figure 2:**
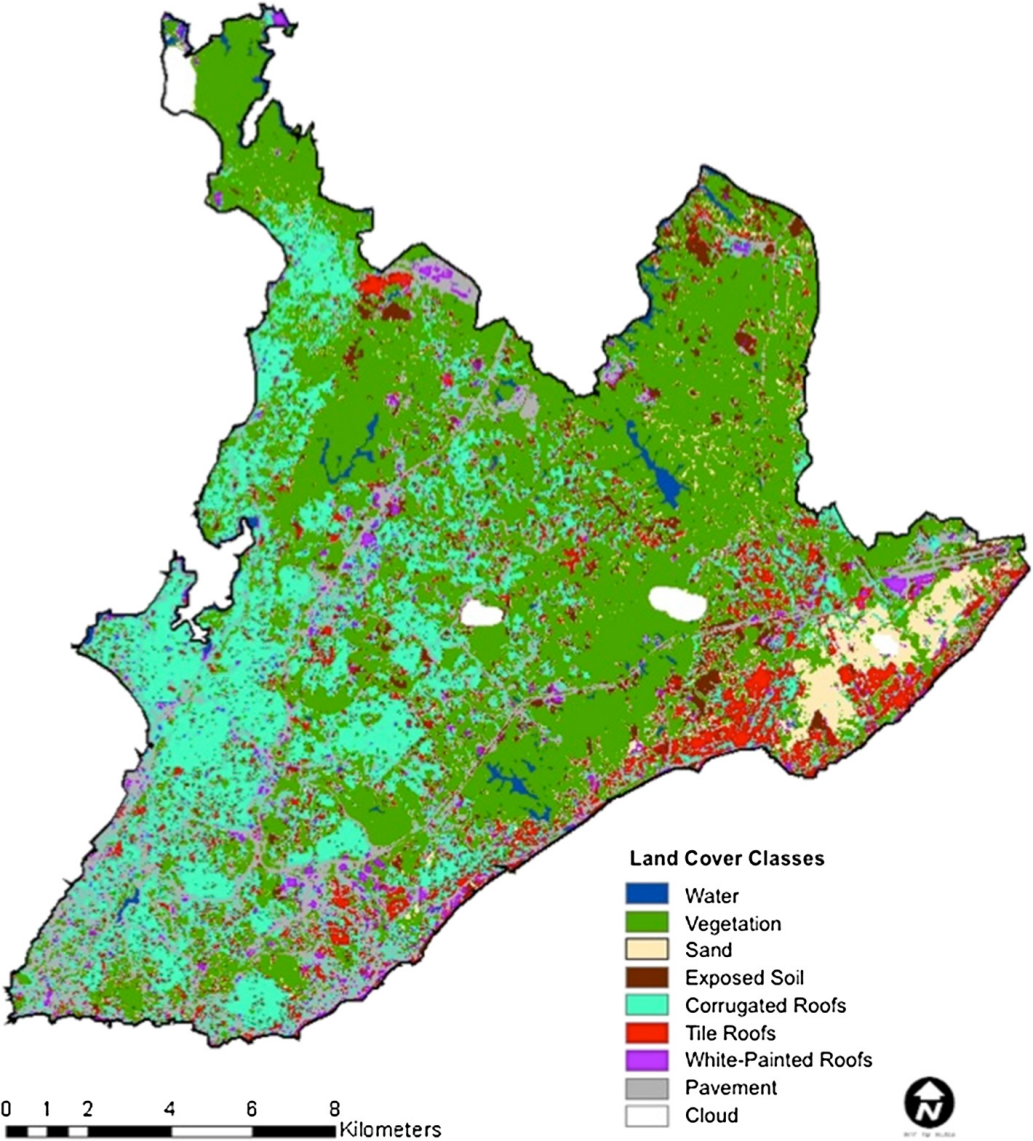
**Land cover classification.** Landsat TM 2002 Land cover classification based on water, vegetation, sand, exposed soil, corrugated roofs, tile roofs, white-painted roofs, pavement, and cloud land covers.

To capture the structural quality of housing listed in the UN-Habitat definition of a slum, we generated a land cover classification of a Landsat TM image acquired on February 24^th^, 2002, based primarily on roof types (Figures 1A and Figure 2). We hypothesized that corrugated roofs could serve as a proxy for poor structural quality of housing because corrugated steel roof tops are a relatively inexpensive material commonly used for roofing among Brazilian slum households. In contrast, red tile roofs and white painted roofs are associated with higher income residential areas and commercial or apartment buildings, respectively. A ‘texture’ layer was also derived using a 3 × 3 pixel window high-pass filter on the red band of the Landsat image. Higher standard deviation of this texture layer was associated with increased edge between buildings and roads, a common feature of higher income areas. In contrast, slum areas were more spatially homogenous. We also mapped pavement, vegetation, water, sand, and exposed soil (Figure 2). We assessed the accuracy of the classification using a fuzzy accuracy assessment, which accounts for intra-pixel heterogeneity (see Methods). The weighted fuzzy accuracy of the classification was 79% with an un-weighted kappa of 0.68, 71% fuzzy weighted user’s accuracy and 86% weighted producer’s accuracy. The classification had a higher level of accuracy for non-urban than urban classes (Additional file 1). The pavement classification had the lowest accuracy of all classes. Importantly, corrugated roofs were rarely classified as either tile or complex roofs (0.012%); the majority of errors in the corrugated roof classification were due to areas that were too heterogeneous to assign to any particular class.

### Canonical correlation analysis of land cover and socioeconomic data

We used canonical correlation analysis to test the hypothesis that land cover metrics were associated with socioeconomic variables (Figure 1B, Table 2). The canonical correlation analysis identified a significant association between the two sets of variables and derived a set of orthogonal dimensions along which the land cover variables were most strongly correlated with the socioeconomic variables. In a dimensionality test, the first three dimensions, or canonical variates, were significant at the 0.05 level using the chi-squared approximation, while the last two dimensions were not (Table 3). The canonical coefficients for the variables in each of the dimensions are shown in Table 4, the canonical loadings – which measure the linear correlation between each variable and their respective canonical variate, are shown in Table 5.

**Table 3:** Significance tests of the canonical variates

**Dimension**	**Canonical variates**	**Wilks lambda**	**Eigen value**	**Approximate chi-sq**	**DF**	**P-value**
**1**	0.586	0.569	0.523	1382.560	25	<0.000
**2**	0.347	0.866	0.137	351.770	16	<0.000
**3**	0.113	0.985	0.013	36.23	9	<0.000
**4**	0.042	0.998	0.002	4.500	4	0.343
**5**	0.010	1.000	0.000	0.220	1	0.636

**Table 4:** Standardized canonical coefficients for the significant dimensions of the canonical correlation analysis

**Variables**	**Significant dimensions**		
	**1**	**2**	**3**
**Socioeconomic**			
Bathroom	−0.449	0.752	−0.034
Water	0.163	−0.132	−0.433
Garbage	−0.108	0.299	−0.109
Income	0.250	−0.241	1.164
Crowding	−1.055	−0.092	0.940
**Land cover**			
Corrugated roof	0.453	−1.767	−0.698
White-painted roof	−0.23	−0.613	0.733
Tile roof	−0.067	−0.5	0.151
Pavement	−0.451	−1.291	−1.205
Texture	−0.051	−0.372	−0.086

**Table 5:** Canonical loadings for the significant dimensions of the canonical correlation analysis

**Variables**	**Significant dimensions**		
	**1**	**2**	**3**
**Socioeconomic**			
Bathroom	0.057	0.944	0.163
Water	−0.128	−0.711	−0.354
Garbage	0.051	0.689	0.037
Income	−0.845	−0.526	0.042
Crowding	0.645	0.245	0.701
**Land cover**			
Corrugated roof	0.559	−0.097	−0.003
White-painted roof	−0.348	−0.116	0.071
Tile roof	−0.067	−0.073	0.037
Pavement	−0.535	−0.013	−0.044
Texture	−0.130	−0.143	−0.002

**Figure 3:**
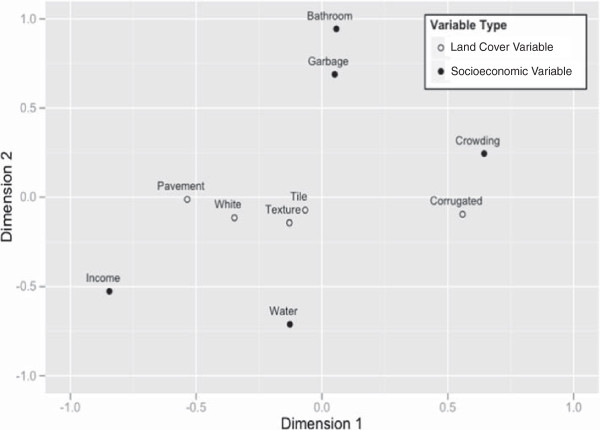
**Socioeconomic and land cover variables in the first two canonical dimensions.** Canonical loadings for each original variable in the first two canonical dimensions. Variables definitions are listed in Table 2.

The first dimension of the canonical correlation captured the strongest association between the socioeconomic and land cover variables. The ordination of census tracts along the first canonical dimension captured a gradient from slum areas (positive loadings) to higher income areas (negative loadings) (Figure 3). This gradient can be inferred from the canonical loadings associated with this dimension: positive for crowding and corrugated roofs, negative for pavement, white-painted roofs and income (Figure 3, Table 5). Based on the canonical coefficients of the land cover variables, corrugated roofs and pavement variables most strongly influenced the first dimension (Table 4). Of the socioeconomic variables, income and bathroom variables had the most influence in the first dimension (Table 4). Based on the canonical loadings, the second canonical dimension was mostly driven by socioeconomic variables such as bathroom, garbage and crowding (positive) and income and water (negative) (Figure 3, Table 5). The majority of households in Salvador have access to sanitation and piped water, which may account for why these variables explain less of the overall variance. Based on this lack of infrastructure, communities that are highly loaded in this dimension are likely newly-squatted areas. The land cover variables did not weigh strongly in this dimension, however dimension 2 still identifies important deprivation characteristics (Table 5). The third dimension explained only a 0.11 of the variance and differentiated high-income areas with high proportion of white-painted (typical commercial areas in Salvador) or tiled roofs from lower-income areas.

### Mapping slum settlements using canonical variates

**Figure 4:**
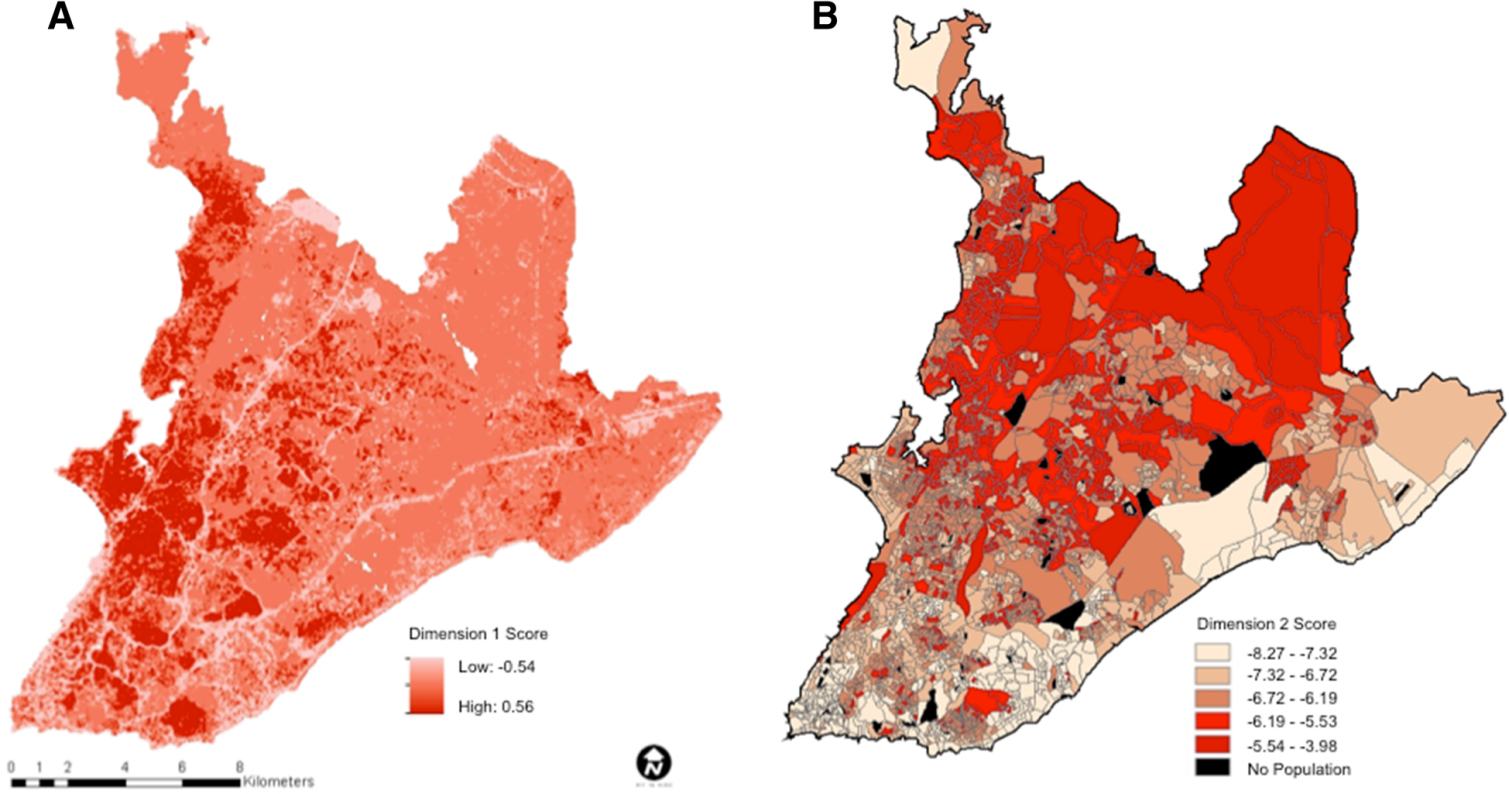
**First and second dimension of the canonical correlation analysis.** Visual representation of the Dimension 1 score mapped at the pixel level **(A)** and Dimension 2 score mapped at the census tract level **(B)**.

As a methodological exercise, we produced a map of slums by integrating the two first canonical dimensions (Figure 1C). The flow diagram for this procedure is exemplified for a single census tract in Additional file 2 and described in detail in the methods section. Because the land cover variables had a strong influence in the first canonical dimension, we mapped the first dimension at the pixel level using a linear combination of the land cover variables, weighted by their respective canonical loadings (Figure 4A). Because the second dimension was not strongly associated with land cover variables, we mapped this dimension at the census tract level using a linear combination of socioeconomic variables weighted by their respective loadings (Figure 4B). The median number of 30 m × 30 m pixels per census tract was 44 pixels (39,138 m^2^), the mean was 123 pixels (110,688 m^2^) and they ranged from 2 to 13,090 pixels (1,490 to 11,784,867 m^2^). Using our approach we were able to increase the spatial resolution of the socioeconomic data incorporated into the final map.

**Figure 5:**
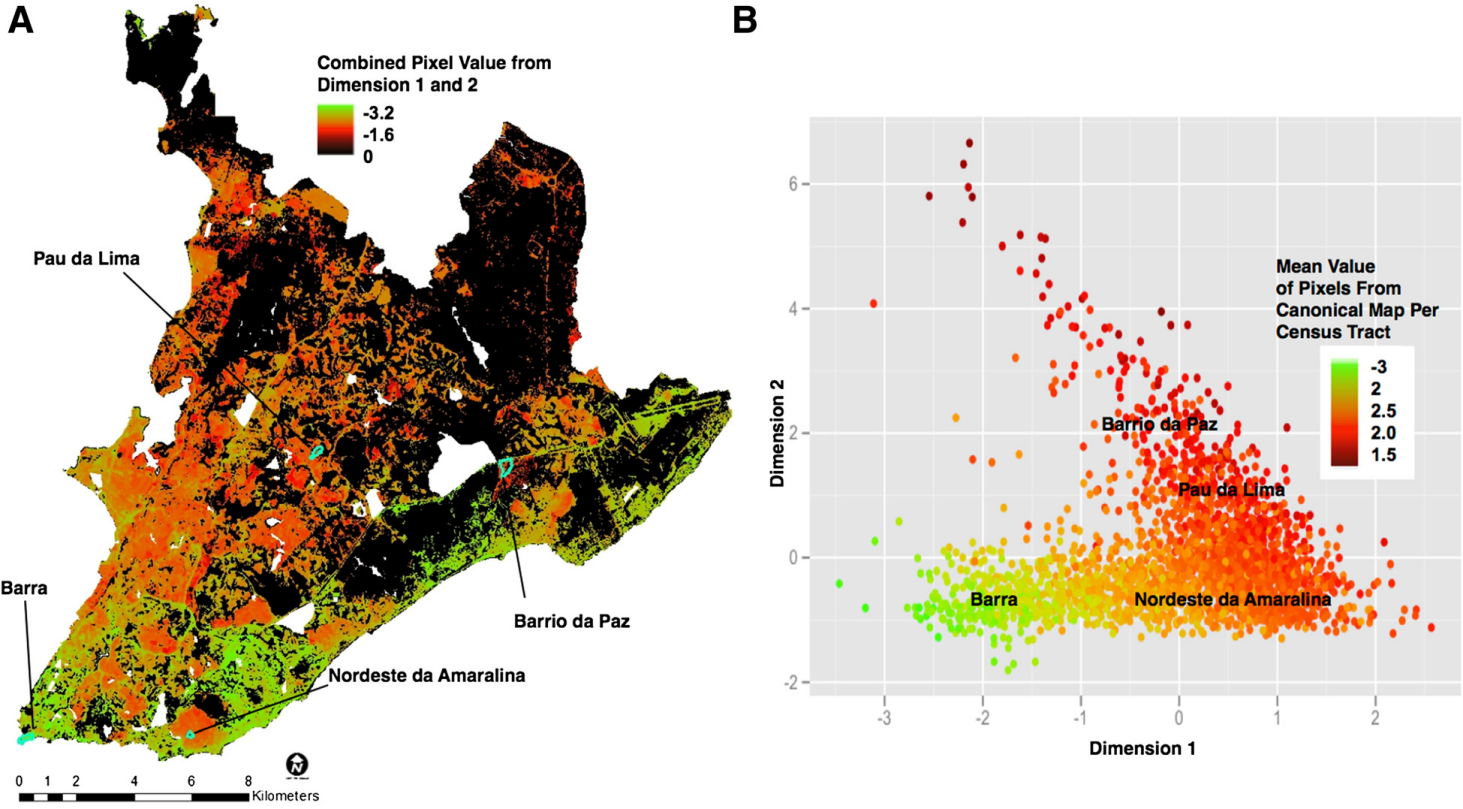
**Mapping in geographic and canonical space. A)** Map of deprivation in Salvador at 30 m × 30 m resolution using combined canonical dimensions 1 and 2. Green areas are at a low risk of slum characteristics, while red indicates a high risk for slum characteristics. The final map was clipped to the impervious surfaces. The geographic location of selected communities representing a range of land cover and socioeconomic characteristics is also represented. **B)** Location of the same communities in canonical space. Canonical scores for dimensions 1 and 2 are shown for all census tracts; the colors correspond to the mean combined dimension 1 and 2 score for each census tract (as represented in **A)**.

We produced the final deprivation map by summing the values of both dimensions for each pixel (Figure 5A), thus incorporating attributes identified in both the first and second dimensions. Additionally, we generated a visual representation of Salvador urban structure in canonical space by plotting the mean pixel value for each census tract derived from the final map (Figure 5B). Canonical scores for all census tracts represented a continuum, with the least developed and lower income areas loaded most positively (red) and more affluent areas were loaded negatively (green) in both dimensions. To assess the position of slum communities in canonical space, we selected three communities that represented different levels of urban slum development and age, as well as a higher-income community for comparison (Table 6, Figure 5). The slum communities loaded highly in dimension one, while Barra, the high-income community, loaded low. Among the three slum communities, the newest area with the poorest infrastructure, Barrio da Paz (settled in 1982) loaded highest in dimension 2, followed by Pau da Lima (settled in 1950) and Nordeste Amaralina, the oldest community, first inhabited in 1629 [20].

**Table 6:** Descriptive statistics for variables used in the canonical analyses for selected communities

**Socioeconomic variables**	**Income**	**Bathroom**	**Water**	**Garbage**	**Crowding**
Barrio da Paz	216.742	0.072	0.814	0.025	0.236
Pau da Lima	163.570	0.157	0.607	0.003	0.152
Nordeste Amaralina	391.503	0.003	0.981	0.000	0.135
Barra	2851.771	0.000	1.000	0.000	0.060
**Land cover variables**	**Corrugated roof**	**White roof**	**Tile roof**	**Pavement**	**Texture**
Barrio da Paz	70.824	0.000	0.014	29.163	16.340
Pau da Lima	21.873	0.000	0.146	34.236	24.790
Nordeste Amaralina	90.361	0.000	0.127	4.756	33.190
Barra	19.469	19.469	0.061	58.406	15.380

## Discussion

We developed a systematic yet flexible approach to model the structure of urban deprivation by integrating socioeconomic and land cover characteristics. Current operational definitions of urban slums do not adequately address the dynamic and heterogeneous nature of slum communities. Our modeling approach generates pixel-level information on levels of deprivation, which reduces the spatial uncertainty in aggregate census-level maps and incorporates natural heterogeneity within urban slums. We found that urban slums were not discrete entities with homogeneously shared characteristics of deprivation and marginalization, but were rather part of an urban continuum. These findings highlight the complexity inherent in these communities and challenge efforts to apply rigid or simplistic classification schemes that aim to draw simple correlations between, for instance, changes in land cover characteristics and improvements in human health outcomes [21].

The results of the canonical correlation analysis confirmed our hypothesis that land cover variables are associated with socioeconomic variables. This association is particularly strong in the first dimension of the canonical correlation analysis, which captures a gradient along both land cover and socioeconomic metrics. The second dimension provides a more nuanced differentiation of slum settlements by identifying communities that lack basic infrastructure, are often newly squatted, and represent heavily marginalized portions of the city. Because these characteristics were not strongly associated to land cover, socioeconomic data weigh most strongly in this dimension. By combining the second dimension with the first dimension, we were able to identify and map the most marginalized areas, which may not have been identified from land cover or socioeconomic variables alone.

Our approach provides a crucially needed improvement in the spatial resolution of deprivation mapping that highlights areas of maximum marginalization. By identifying land cover proxies for urban deprivation, we were able to increase the map resolution from the census tract level to the 30 m pixel level, thus capturing within slum heterogeneity and reducing the spatial uncertainty of census-level maps. This higher spatial resolution provides an essential tool for programs aiming at improving access to services to the most marginalized populations. In particular, access to health care could be significantly improved through targeted immunization campaigns and triage of services and hospitals, improved access to education, better design of refuse collection and transportation, and development of community wide initiatives to increase leisure and exercise space [5,22-28]. The more specific targeting interventions achieved by disaggregating census tract data to the pixel level would improve upon previous census level mapping approaches, which may not adequately capture internal heterogeneity and may overestimate the size and scope of urban slums [14-16].

Urban slum settlements within Salvador are not discrete entities with homogenous characteristics, but are rather part of an urban continuum of deprivation levels, as illustrated in the canonical plot (‘canonical space’). In geographic space, slum communities are more clearly defined in the eastern parts of the city than in western Salvador, where there is a larger range of deprivation levels. The representation of the census tracts in canonical space provides a snapshot of the urban structure in Salvador in the early 2000s. Urban slums evolve from squatter settlements to de facto, well-established communities as they gradually receive infrastructure [2,28]. The canonical modeling approach distinguishes slum communities at different stages of their development and evolution, as illustrated by the position of the four Salvador communities in the canonical plot (Figure 5). By examining changes in this structure over time, we can describe community transitions as development or public health policies and interventions are implemented.

In addition to applications to city-wide infrastructure projects, the proposed approach can serve as a tool to guide policies aimed at changing the built and social environments of poor/informal areas of cities. The proposed approach can be used by municipal/national policy makers, urban planners and public health officials to guide interventions and measure the social and physical impacts of policies focused on access to health, transport, housing, social inclusion and environmental/climate change vulnerability. By tracking a community’s progression across canonical space we can quantitatively monitor the impacts of these policy interventions and assess the evolution of urban slums. Furthermore, our approach generates socioeconomic and land cover metrics that can be used as parameters or to validate mechanistic models of urban expansion.

Despite our approach’s flexibility, the constraints of the canonical correlation analysis and the resolution of our data limited the accuracy of the overall model. Unlike other techniques currently used to map urban slums [9-14,16,29] our method does not require expensive very high resolution imagery or specialized object-oriented classification software. Accuracy could be improved by using higher resolution data when available, which can easily be incorporated in this approach. High-resolution imagery would likely only be affordable for smaller area studies of neighborhoods of particular interest. While we were able to accurately describe socioeconomic characteristics of urban slums, some potentially important variables such as insecure access to tenure were not included in this model. There were also outlier communities with low infrastructure despite higher levels of income. These areas are likely squatter settlements among higher income census tracts, and overall account for less than 0.014% of the total census tracts considered. The selections of variables from the Brazilian census used in the canonical correlation analysis were also constrained by the linearity requirement.

While this approach was applied specifically to Salvador, Brazil, it is flexible enough to incorporate a wide variety of data sets that may be critical to other regions of interest. Our objective was to develop a low-cost method/model that might be applied in other urban environments where there are data collection challenges/gaps, which is the norm for most urban informal settlements in the global south. Our approach does require census data, which could limit its generalizability to other regions. However, the quality of census data throughout the world is increasing in major cities like Delhi, Accra, and in cities throughout Brazil. The specific associations between the socioeconomic and land cover variables, while specific to Salvador, can with further validation be used in other urban slum environments, particularly in Brazil. While we based our analysis loosely off the UN-HABITAT definition, our approach allows for the incorporation of various socioeconomic or land cover variables that can be modified based on site-specific attributes, such as insecure access to tenure or thatched rooftops instead of corrugated roofs as a proxy for poor quality of housing.

## Conclusions

We created a model that captured the heterogeneous and dynamic nature of urban deprivation in Salvador, identified communities that are most at risk, and created a framework to examine the effects of policy decisions such as slum upgrading. Post-hoc evaluations of slum upgrading are rare and program evaluations tend to only study narrow intervention objectives, such as changes in the incidence one disease, family income, household expenditure or access to service. Our method offers an analytic process for tracking broader changes in the size, shape and extent - or ‘structure’ - of slums.

This approach is synoptic and provides a cost-effective examination of large areas, which can be comparable across regions. It has limited subjectivity, compared with maps purely reliant on local expertise. Local expertise can however be readily incorporated during the process of identifying critical variables to be considered or for mapping specific focus areas at higher resolution. Thus, our approach calls for continued research partnerships between slum dwellers and professionals to ensure that our maps are realistic, locally meaningful, while cost effective. Unlike other deprivation scoring methods, this method allows for the incorporation of remotely sensed data along with socioeconomic data, providing a method to map deprivation at fine resolution.

Mapping using canonical correlation analysis can additionally be used in a variety of applications beyond slum characterization, where it is necessary to ascertain complex relationships between the land cover and the socioeconomic environment, and monitor the effects of policy decisions.

## Methods

### Study site

Salvador is located in the State of Bahia, Brazil. It was the first capital of Brazil and today is the largest city in the Northeast, and the third most populous city in the country. Currently 3 million people live in Salvador, and in 2000 an estimated 60% of the population lived in slums, according to a census level ‘poverty map’ (Mapa de Pobreza e Desigualdade) developed by the IBGE [17,28,30,31].

### Socioeconomic variables

The socioeconomic variables were derived from the 2000 IBGE census [31]. We selected variables that approximated the socioeconomic characteristics of urban poverty in Salvador and Brazil [5,32,33] as well as characteristics that approximated the salient characteristics of the UN-Habitat definition of urban slums (Table 1). Canonical correlation analysis requires a linear relationship between the socioeconomic and land cover variables, which limited the final set of variables used to those in Table 2.

The variables selected are proxies for sanitation, crowding, income, and access to water. The proportion of households with piped water was used as a proxy for inadequate access to safe water. The proportion of households without bathrooms was used as a proxy for inadequate access to sanitation. The proportion of households with more than 6 residents per house was used as a proxy for overcrowding. The UN-Habitat group defines overcrowding as having ≥ 3 people sharing a room. Since many homes in slum areas have few rooms, we chose a threshold criteria of 6 or more residents per house to approximate the UN-habitat overcrowding definition, e.g. for a household with 2 rooms, 6 residents would meet the overcrowding definition [2]. Average monthly income was used to approximate income. Unlike many other slum communities, most urban dwellers in Brazil operate in the formal economy, and are therefore able to provide accurate estimates of income, thus providing an acceptable measurement of socioeconomic status, compared to those operating primarily in the informal economy. While income may still be prone to reporting bias [34], it has been shown to be associated with urban health in Salvador [5,28], and was therefore included in the canonical correlation analysis.

### Land cover variables

A Landsat Thematic Mapper (Landsat TM 5) scene, processing level 1, 30 m pixel resolution, acquired on February 24th 2002 was used for this study. The image was georeferenced to the Universal Transverse Mercator (UTM) zone 24S coordinate system.

A land cover classification was generated based on the following land cover classes: corrugated roofs, tile roofs, white-painted roofs, pavement, vegetation, water, sand exposed soil and clouds. Training data for each land cover type was collected from 55 field sites throughout Salvador during June 2011. During the field visits, observers verified homogeneity of features of at least 60 m × 60 m (2 × 2 pixels) and recorded them on a 2002 georeferenced orthoimage. If land cover changes had occurred at the site based on visual comparison to the orthoimage, the site was not used as a training area. These data were used to train a maximum likelihood classification algorithm in ENVI 2.0 *(Exelis Visual Information Solutions, Boulder, Colorado)*. To assess the accuracy of the classification, we generated a random selection of 543 homogenous 60 m × 60 m (2 × 2 pixel) areas from the land cover classification and assessed their concordance with the same area in the 2002 orthoimage. We assigned a score based on their concordance using a modified fuzzy accuracy method [35-37] as follows: (1) no match; (2) < 50% of the observed class present in the 2 × 2 window; (3) between 50% and 75% of the class present; (4) between 75% and 90% of the class present and (5) > 90% of the class present. The overall fuzzy accuracy, fuzzy producer’s and user’s accuracies, kappa statistic, and weighted fuzzy accuracies were calculated [36,37]. If the 2 × 2 pixel area was too heterogeneous to assign to a class, pixel areas were considered as “heterogeneous” in the confusion matrix.

### Canonical correlation analysis

Canonical correlation analysis is a multivariate statistical technique that estimates the correlation between two sets of variables [38]. Canonical correlation analysis aids in the study of the interrelationship between multiple dependent and multiple independent variables, in this case the socioeconomic variables and the land cover variables. For this analysis we focused on analyzing the canonical variates, canonical coefficients and canonical loadings. Canonical variates, or canonical dimensions, are orthogonal linear combinations of the dependent and independent variables that maximally correlate with each other. To determine how many dimensions were significantly correlated, we used a chi-squared analysis [38]. The canonical coefficients, also called canonical weights, measure the contribution of each variable to the canonical variate [38]. A small weight may mean either that the corresponding variable is irrelevant in determining a relationship or that it has been partialed out of the relationship because of a high degree of multicollinearity [38]. Canonical loadings are therefore estimated to measure the independent influence of each variable irrespective of other variables considered. Canonical loadings measure the simple linear correlation between each variable and their respective canonical variate (the variance that the observed variable shares with the canonical variate). The higher the canonical loading the more it contributes to the respective canonical variate.

The variables used in the canonical correlation are summarized in Table 2. Prior to performing the canonical correlation analysis, we employed two types of data transformations to improve the linearity of our variables. The income variable was log transformed, and since the remaining variables were both 0- and 1-inflated, they were transformed using the arcsine square root transformation. We performed the canonical correlation analysis on the transformed data.

### Mapping using canonical coefficients and variates

We used the outcomes from the canonical analyses to produce a 30 m × 30 m resolution map of deprivation across Salvador. A detailed flow diagram of the processing steps for a sample census tract is provided in Additional file 2, with working steps labeled as a - i. The land cover variable inputs for the canonical correlation analyses were the proportions of the different land cover types in each census tract (Additional file 2 working step a) and the standard deviation of the texture layer (Additional file 2 working step b). To produce a pixel level map, we calculated the same metrics (proportion of each land cover types and texture) but in a 3 × 3 pixel moving window rather than the whole census tract. We first extracted each land cover type from the land cover classification (Additional file 2 working step c) and calculated the proportion of pixels of each class in a 3 × 3 pixel window (Additional file 2 working step e). We also calculated the standard deviation of the texture (Additional file 2 working step d) in a 3 × 3 moving window using the focal statistics procedure in ArcGIS (ArcGIS 10.1 Environmental Systems Resource Institute, Redlands, California) (Additional file 2 working step f). These layers were weighted by the canonical loadings from the first dimension and added to generate a score for dimension 1 (Additional file 2 working step g):

(1) $$\begin{array}{c} \text{Dimension}\;1\;\text{score}=\left(0.559\right)\text{CO}+\left(0.348\right)\text{WP}\\ \;\;\;+\left(-0.067\right)\text{TR}\\ \;\;\;+\left(-0.535\right)\text{PA}\\ \;\;\;+\left(-0.130\right)\text{TX} \end{array}$$

where CO: % of corrugated roofs in a 3×3 window around the pixel, WP: % white painted roofs, TR: % tile roofs, PA: % pavement, TX: mean texture in a 3×3 window around the pixel.

Since the second dimension was not strongly associated to land cover variables, we mapped the second dimension at the level of the census tract using the dimension 2 canonical loadings for the socioeconomic variables (Additional file 2 working step h). We used the polygon to raster function in ArcGIS to map the socioeconomic information at the pixel level, and applied the following map algebra equation:

(2) $$\begin{array}{c} \text{Dimension}\;2\;\text{score}=\left(0.944\right)\text{BA}+\left(‒0.711\right)\mathrm{WA}\\ \;\;\;+\left(0.689\right)\text{GA}\\ \;\;\;\;+\left(‒0.526\right)\mathrm{IN}+\left(0.245\right)\text{CR} \end{array}$$

where BA: % houses with bathroom, WA: water, GA: garbage, IN: income, CR: crowding.

Pixel scores for each of the dimensions were weighted by their corresponding canonical variates (Table 3) and added to generate a visual representation of the two dimensions at the pixel level (Additional file 2: Figure S2 working step i):

(3) $$\begin{array}{l} \text{Combined}\;\text{Dimensions}\;1\;\text{and}\;2\;\text{scores}\\ \;=\left(0.586\right)D1+\left(0.347\right)D2 \end{array}$$

where D1: Dimension 1 score, D2: Dimension 2 score.

To produce the final map, we masked all non-urban pixels (water, vegetation, sand, exposed soil). The third dimension was not included in the map as it accounted for the least amount of variability and did not appear to identify characteristics of slums communities.

## Competing interests

The authors declared that they have no competing interests.

## Authors’ contributions

KPH, KCS, AIK, MADW designed the study. KPH, MGR, and FC created databases used in the study. KPH and FC performed field work. KPH, KCS, and MADW performed the data analysis. KPH wrote the manuscript. All authors read, made substantial contributions to and approved the manuscript.

## Supplementary Material

Additional file 1 Fuzzy confusion matrix. Click here for file

Additional file 2 **Flow diagram of methodology for a representative census tract in Salvador.** Each working step is labeled as a – i. Working step a depicts the land cover classification from the original Landsat TM imagery. Working step b depicts using the original Landsat TM imagery to perform the texture analysis. Working step c depicts the extraction of the individual impervious land-cover types into separate rasters. Working step d depicts the high-pass filter of the red Landsat TM band to detect edge characteristics. Working step e depicts calculating the proportion of each impervious land-cover type within a moving 3 × 3 pixel window. Working step f depicts calculating the standard deviation of the texture layer within a moving 3 × 3 pixel window. Working steps g and h represent using the canonical loadings as weights to create visual displays of dimensions 1 and 2 (Figure 4A and Figure 4B). The final working step i combines the two dimensions weighted by the canonical variates creating the final map depicted in Figure 5. Click here for file
