# Supplementary material for: Urban slum structure: integrating socioeconomic and land cover data to model slum evolution in Salvador, Brazil
Source: Int J Health Geogr. 2013 Oct 20;12:45. doi: 10.1186/1476-072X-12-45 (PMC3924348; doi:10.1186/1476-072X-12-45)
Supplement: Additional file 2 — Flow diagram of methodology for a representative census tract in Salvador. Each working step is labeled as a – i. Working step a depicts the land cover classification from the original Landsat TM imagery. Working step b depicts using the original Landsat TM imagery to perform the texture analysis. Working step c depicts the extraction of the individual impervious land-cover types into separate rasters. Working step d depicts the high-pass filter of the red Landsat TM band to detect edge characteristics. Working step e depicts calculating the proportion of each impervious land-cover type within a moving 3 × 3 pixel window. Working step f depicts calculating the standard deviation of the texture layer within a moving 3 × 3 pixel window. Working steps g and h represent using the canonical loadings as weights to create visual displays of dimensions 1 and 2 (Figure 4A and Figure 4B). The final working step i combines the two dimensions weighted by the canonical variates creating the final map depicted in Figure 5. [file 1476-072X-12-45-S2.pdf]

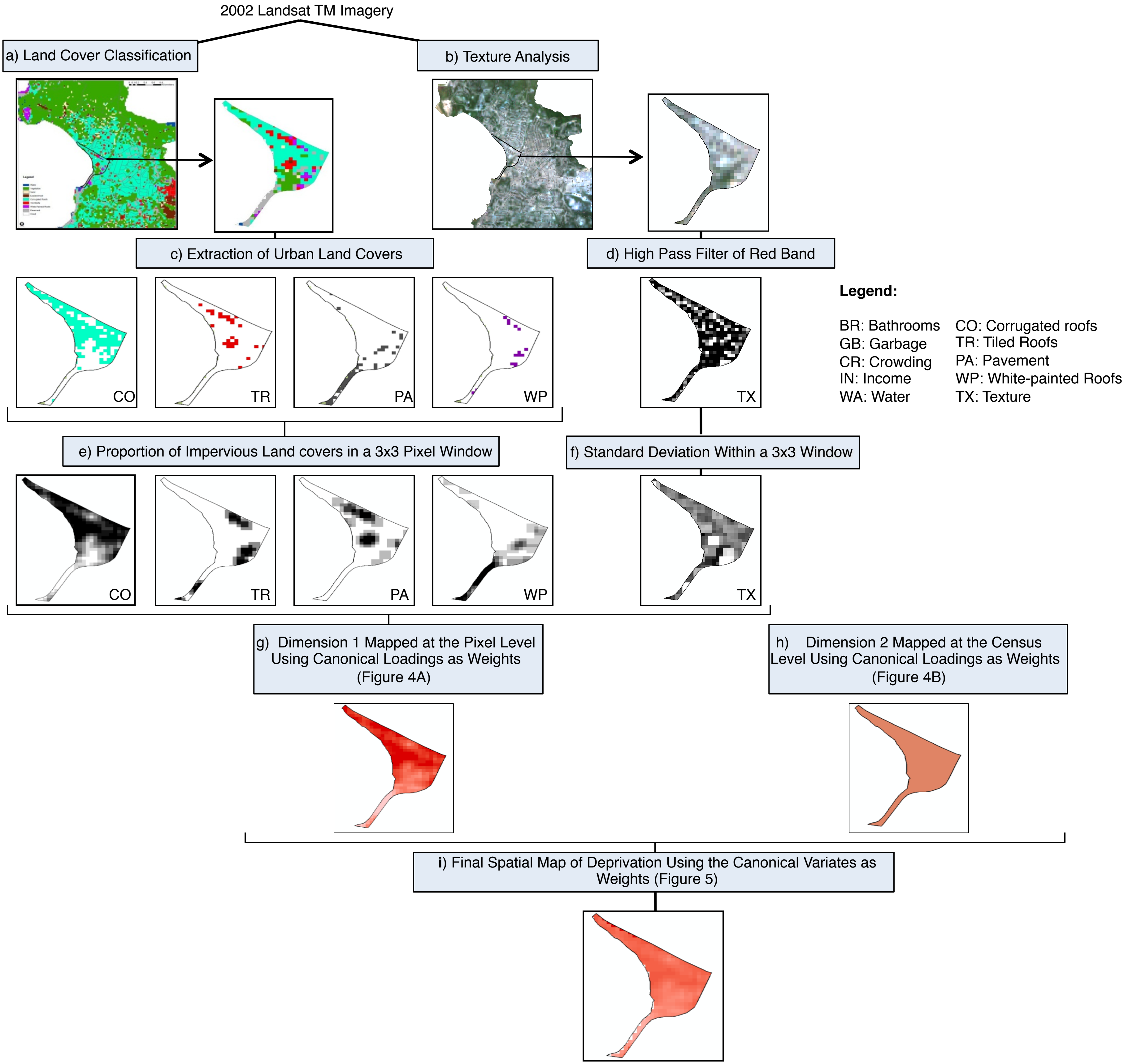
2002 Landsat TM Imagery
a) Land Cover Classification
b) Texture Analysis
c) Extraction of Urban Land Covers
d) High Pass Filter of Red Band
CO
TR
PA
WP
TX
Legend:
BR: Bathrooms
GB: Garbage
CR: Crowding
IN: Income
WA: Water
CO: Corrugated roofs
TR: Tiled Roofs
PA: Pavement
WP: White-painted Roofs
TX: Texture
e) Proportion of Impervious Land covers in a 3x3 Pixel Window
f) Standard Deviation Within a 3x3 Window
CO
TR
PA
WP
TX
g) Dimension 1 Mapped at the Pixel Level Using Canonical Loadings as Weights (Figure 4A)
h) Dimension 2 Mapped at the Census Level Using Canonical Loadings as Weights (Figure 4B)
i) Final Spatial Map of Deprivation Using the Canonical Variates as Weights (Figure 5)
